# Modeling the predictive role of music teachers’ job commitment and optimism in their sense of self-efficacy

**DOI:** 10.3389/fpsyg.2023.1291443

**Published:** 2023-11-09

**Authors:** Yadian Du

**Affiliations:** Department of Music, Xinxiang University, Xinxiang, China

**Keywords:** music education, teacher job commitment, academic optimism, teacher self-efficacy, SEM

## Abstract

**Introduction:**

Teachers’ psychological factors have been argued to influence various aspects of music education. However, scant research has explored the psycho-affective aspects of music teachers’ work. To solve this shortage of research, this study examined the predictive role of Chinese music teachers’ job commitment and academic optimism in their self-efficacy.

**Methods:**

To this end, 340 music teachers from four universities in Henan Province completed an online survey including three questionnaires.

**Results:**

The results of structural equation modeling (SEM) and regression analysis demonstrated that music teachers’ self-efficacy could be positively and significantly predicted by their optimism and job commitment.

**Discussion:**

The study provided implications for music education to pay further attention to the psychology of teaching in this field. Finally, directions for further research are presented to scholars, who are interested in the psycho-emotional side of music education.

## 1. Introduction

Music teaching is an important profession all around the world given its significance in social life (Kandemir, 2019). It requires many psychological constructs to succeed (Wang et al., 2022; Pan, 2023; Pan et al., 2023; Wang and Derakhshan, 2023). One such construct is teacher commitment, which has been considered as a pivotal issue in effective music education (Türk and Korkmaz, 2022). Teachers’ commitment refers to their affective bond with their job (Shukla, 2014) that shapes their identity and energy (Bakker and Leiter, 2010). It influences teachers’ job satisfaction, retention, and perceived responsibility and loyalty (Celep, 2000; Klassen and Chiu, 2011). The level of teachers’ commitment directly affects their rate of attrition, resignation, dissatisfaction, and demotivation (Mulkeen et al., 2007). In arts education, like other disciplines, teacher commitment holds significance and meaning in that it leads to a positive learning atmosphere, student growth, and academic success (Halim and Marzidi, 2023). Research shows that different demographic and psycho-affective factors influence teachers’ perceived commitment (Moses et al., 2016).

A construct that may correlate with teachers’ commitment is academic optimism (Kurz, 2006). Optimism is a concept that flourished with the advent of positive psychology (PP). It refers to one’s perceived hope, obligation, and positive attitude toward his/her life and job (Seligman, 2006). Optimism is critical to academic performance and success because optimistic teachers typically focus on strengths rather than weaknesses, pay attention to the bright side, and try to find solutions rather than succumb to the challenges (Pathak and Lata, 2018). Teachers’ optimism creates positive beliefs and learning environments for teachers and students (Hoy et al., 2006). Empirical studies support the contribution of teacher optimism to many other positive factors such as resilience, well-being, rapport, classroom engagement, self-confidence, and job commitment (Hoy and Tarter, 2011; Lu, 2021; Dong and Xu, 2022; Wang et al., 2022; Pan et al., 2023). However, the predictive role of teachers’ optimism and commitment in their sense of self-efficacy has rarely been empirically explored in arts education. Most of the existing studies are limited to general education, psychology, and second/foreign language education. As arts education is essential to students’ growth, teachers should be skilled and certain of their abilities to provide meaningful arts instructions (Lummis et al., 2014).

To make this happen, teachers’ self-efficacy should be enhanced in arts education as this construct determines their future actions (De Vries, 2013; Derakhshan and Fathi, 2023). The concept of self-efficacy refers to one’s ‘perceived operative capability’, which he/she gains a desired level of success. Likewise, teachers’ self-efficacy concerns their beliefs in their capability to influence students’ learning (Garvis and Pendergast, 2010). Some studies in arts education research argued that teachers’ self-efficacy beliefs shape classroom engagement, digital technologies incorporation, and attitudes toward teaching (Garvis, 2009; De Vries, 2013; Lummis et al., 2014; Saudelli and Ciampa, 2016). However, there is a dearth of research on the interplay of teacher commitment, optimism, and self-efficacy in this line of thinking. Without being a committed teacher, who is optimistic and hopeful of his/her career, it is difficult to be perceived as an efficacious arts teacher. To shed some light on this interaction, the present study aimed to unravel the predictive power of Chinese music teachers’ commitment and optimism in their self-efficacy. In the literature, there is a lack of predictive research on teacher emotions in music education. By focusing on such a gap, the present study may expand the PP landscape from educational psychology to music education.

## 2. Literature review

### 2.1. Teachers’ job commitment

**Figure 1:**
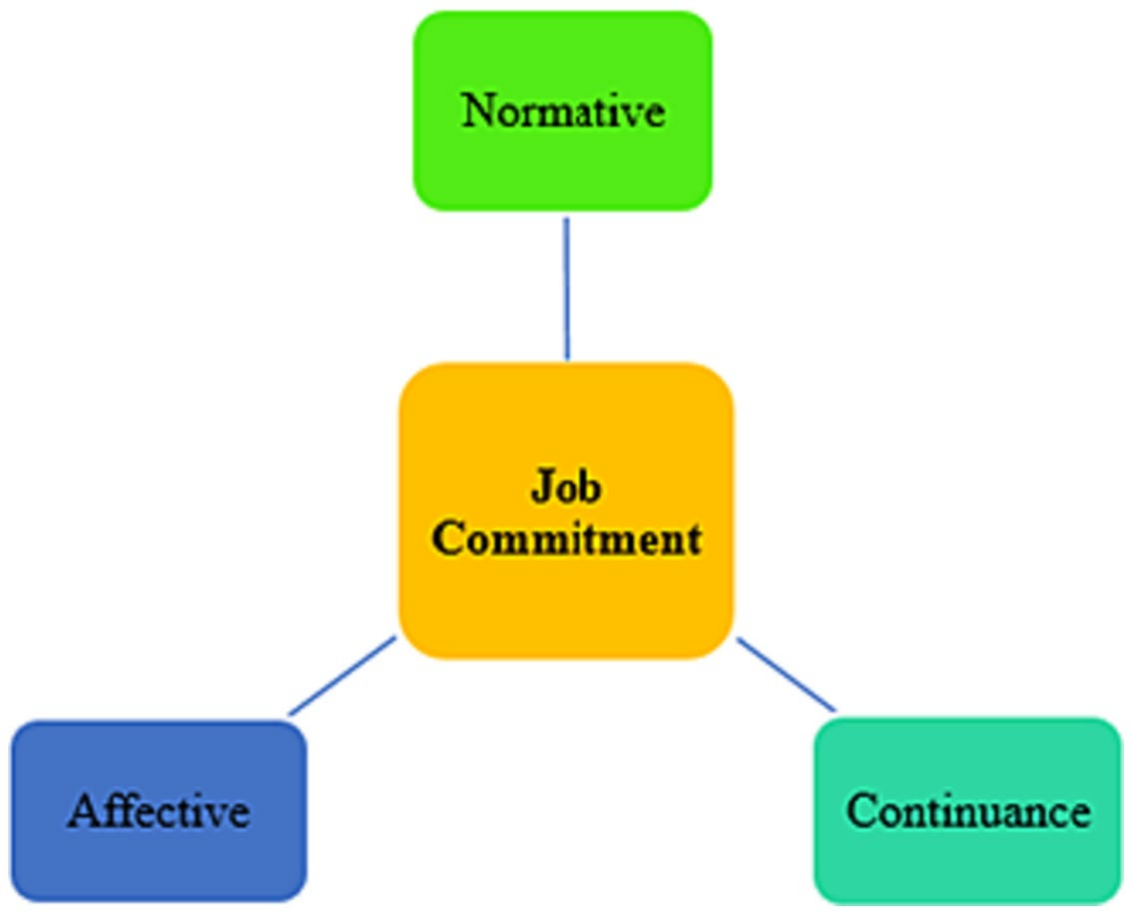
The dimensions of job commitment.

The concept of teacher commitment concerns an instructor’s perceived dedication, enthusiasm, and motivation toward his/her job and students (Halim and Marzidi, 2023; Zhi et al., 2023; Zhi and Wang, 2023). It is a type of psychological attachment to a significant object that bears meaning and importance to a person (Somech and Bogler, 2002). Committed teachers have a strong rapport with pupils, schools, and the teaching profession (Cohen, 2000). They also invest more time and energy in creating attractive classes for students to artistically grow (Lee et al., 2022). Commitment is a multi-dimensional factor involving three components affective, continuance, and normative commitment (Meyer et al., 1993). The first dimension concerns the emotional connection and attachment that a person develops in relation to a profession (Figure 1). Continuance commitment refers to a person’s financial analysis and the cost he/she should pay to leave a job. Hence, he/she remains in the position owing to such challenges and possible penalties.

As the third dimension, normative commitment pertains to one’s moral obligation, allegiance, and faithfulness to a job that prevents him/her from leaving the work. Teacher commitment is crucial for an effective instruction because, without affection for their job, teachers cannot direct education effectively (Crosswell and Elliott, 2004). Teacher commitment is influenced by several individual, contextual, internal, and external factors in academia (Pan, 2023). This is in line with social exchange theory (SET), which argues that teacher commitment is affected by an array of intrinsic and extrinsic factors related to workplaces (Miao et al., 2014). According to SET, individuals’ commitment level is the outcome of their perceived sense of support and community at work. Other than demographic and background factors, teachers’ commitment has been claimed to be affected by internal and psycho-emotional factors (Al-Jabari and Ghazzawi, 2019). Two intrinsic variables that may correlate with music teachers’ sense of job commitment are optimism and self-efficacy, as explained in the subsequent sections.

### 2.2. Academic optimism

**Figure 2:**
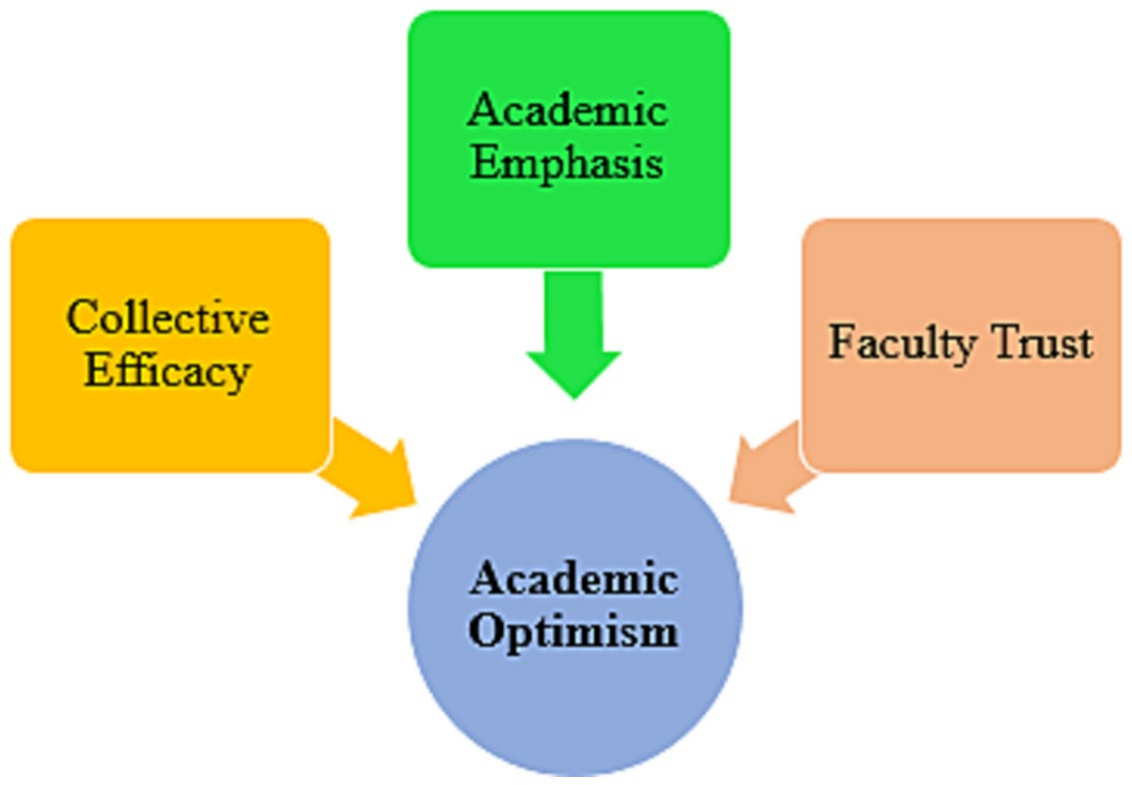
The dimensions of teacher academic optimism.

As a concept derived from PP, optimism refers to a person’s expectation and mood of a future event regarding it as desirable and favorable (Carver and Scheier, 2002). It deals with an inspirational, affective, and psychological position that a person has toward the future occurring (Lu, 2021). Optimistic individuals look on the bright side even when there are difficulties. In teaching, optimism concerns teachers’ intrinsic inclination to have faith in their skills to bring about learning and experience good events rather than troubles and failures (Hoy et al., 2008). As shown in Figure 2, teacher academic optimism has three dimensions of academic emphasis, collective efficacy, and faculty trust, which highlight teachers’ strengths and engender positive outcomes in education (Hoy et al., 2006). As the first dimension of optimism, academic emphasis points to teachers’ behaviors and beliefs in providing an optimistic learning atmosphere to foster learning and academic success (Hoy and Miskel, 2013).

Moreover, collective efficacy concerns teachers’ confidence in their skills to run instruction efficiently and produce achievement in learners. As the third dimension, faculty trust pertains to teachers’ faith in students and parents to be given a role to play in the learning process (Hoy et al., 2006). The construct of optimism is also theoretically founded by Seligman’s (2006) theory of academic optimism and Bandura’s (1986) social cognitive theory (SCT). The former theory underscores the role of positive outlooks of the future in producing and maintaining positive outcomes, while the latter posits the dynamic impact of individual experiences, the actions of others, and environmental factors on one’s learning. These theories underlie this study because academic optimism is an intrinsic, individual factor that is shaped by the mutual interaction of self, others, and social context. It is dynamic and affected by several factors two of which include commitment and self-efficacy. These two factors are supported by the individual basis of academic optimism posited in PP, optimism theory, and SCT.

### 2.3. Self-efficacy

The construct of self-efficacy dates back to the theory of self-regulation, which argues that a person’s beliefs in his/her abilities produce actions that lead to outcomes expected (Bandura, 2001). The aim of self-efficacy is to change one’s beliefs in abilities instead of actual abilities (Bandura, 1997; Han and Wang, 2021). Simply defined, self-efficacy is a perceived operative competence that a person holds of self in a job (Bandura, 1997). In the context of teaching, self-efficacy refers to teachers’ faith in their own abilities to successfully teach students and make them learn the subject (Garvis and Pendergast, 2010). Self-efficacy is critical to education in that self-efficacy beliefs shape one’s future actions and task performance (Skaalvik and Skaalvik, 2007). According to Bandura (1997), teacher self-efficacy is affected by four elements of *mastery experiences* (experiences shaped in teachers due to students’ improved learning and outcomes), *vicarious experiences* (experiences obtained by watching others implementing a task successfully), *verbal persuasion* (feedback that a teacher receives about his/her instruction from others), and *physiological arousal* (teachers’ psycho-affective responses to teaching events).

Teacher self-efficacy draws on SCT and pertains to a personal analysis of the teaching task in light of one’s capacities (Tschannen-Moran et al., 1998). As put by Bandura (1997), individuals with perceived capability to accomplish certain tasks make more attempts to successfully do the tasks. He regards self-efficacy as a regulatory mechanism that shapes various cognitive, motivational, affective, and selection processes in teachers that are pivotal for learning. It is contended that teachers with greater self-efficacy are more confident in their abilities to help all students learn (Bandura, 1997). Teacher self-efficacy is context-specific and affected by different factors as posited by Bandura’s (1997) triadic reciprocal causation model. The model argues that personal factors, behaviors, and environmental factors interact in the class to determine human agency and practice. Teachers’ optimism and commitment are two personal factors that may play a role in this triadic model, which has received scant attention in arts education research.

### 2.4. Previous studies

The psychological foundations and implications of arts education have been cogently stressed in the literature (Grum and Grum, 2016; Mannopovna, 2019). Inspired by such a perspective, different studies focused on the conceptualization and measurement of psycho-affective variables in this field (Jin and Ye, 2022). Teacher commitment is among the variables, which has been considered significant in teachers’ organizational practices (Imran et al., 2017). Previous studies show that teacher commitment correlates with teachers’ sustainable motivation, turnover intentions (Payne and Huffman, 2005), professionalism (Shukla, 2014), and job satisfaction (Thawda and Simon, 2022). Moreover, Halim and Marzidi (2023) conducted a quantitative study in Malaysia with 190 arts teachers and found their commitment strongly correlated with their psychological factors and creativity. Teachers’ commitment has also been found the fuel behind teachers’ practices and attitudes toward their work (Türk and Korkmaz, 2022). In another recent study, Pan (2023) examined the mediating impact of teacher preparedness and professional learning on Taiwanese teachers’ commitment. In the end, she reported a positive correlation among the variables. Another construct that may interact with teachers’ commitment is optimism (Çoban and Demirtaş, 2011). In this regard, Kurz (2006) drew on PP and SCT and explored the relationship between teachers’ optimism and commitment in Ohio. The results showed that teachers’ optimism and positive beliefs were significantly related to their job commitment. Furthermore, Siddique et al. (2023) carried out a correlational study in Pakistan on 61,762 teachers and found a positive and significant relationship between teacher academic optimism and commitment. The factorial structure of academic optimism has also been the center of some studies (Hoy et al., 2008; Hoy and Miskel, 2013; Dai and Wang, 2023).

Another line of research on teacher optimism has focused on its effects on teachers’ perceived resilience, well-being, rapport, classroom engagement, self-confidence, and job commitment (Hoy and Tarter, 2011; Lu, 2021; Dong and Xu, 2022; Hu and Wang, 2023; Wang et al., 2023). However, the joint influence of teacher commitment and optimism on arts teachers’ work-related variables has remained under-researched. A possible construct, which can be predicted by the interplay of teacher commitment and optimism is self-efficacy. Teacher self-efficacy of arts teachers has been claimed to lead to positive engagement in the classroom (Lummis et al., 2014). In another study conducted in Australia, de Vries (2013) found generalist teachers’ self-efficacy beliefs significant in shaping their pedagogical practices and beliefs about teaching arts and music. The mediating role of technology and digital literacy in arts teachers’ self-efficacy has also been proved by Saudelli and Ciampa (2016), who ran an ethnographic study on the impact of technological pedagogical content knowledge (TPACK) on teachers’ self-efficacy in Canada. Additionally, Garvis (2009) tested the contribution of self-efficacy training on arts teachers in Queensland and argued that teachers attended the course could regulate their behaviors and beliefs about teaching more positively. As the review of the existing literature revealed, despite the fact that self-efficacy is an important determinant of both commitment and optimism (Hoy et al., 2006; Skaalvik and Skaalvik, 2007), few empirical studies have been conducted on the interplay between teacher commitment, optimism, and self-efficacy. Furthermore, the earlier studies on job commitment, optimism, and self-efficacy have separately focused on the conceptualizations, measurements, and correlations of these variables with other factors. However, their interaction has been somewhat neglected in the context of art education, so further empirical research on this topic is needed.

### 2.5. Research question and hypothesis

To bridge the gaps in arts education research, this quantitative study seeks to see whether teachers’ commitment and optimism could predict their self-efficacy beliefs. In line with this purpose, the following research question and hypothesis will be addressed:

RQ: How much variance in music teachers’ sense of self-efficacy can be predicted by their job commitment and optimism?

*H_0_*: Job commitment and optimism can not significantly predict teachers’ self-efficacy.

## 3. Methods

### 3.1. Participants

In this study, a sample of 340 Chinese music teachers participated. They were practicing teachers from both genders (males = 48, females = 292). The age of the respondents varied from 20 to 60 years old (*M* = 28). They were graduates of musicology (i.e., the study of all aspects of music across cultures and periods), pedagogy (teaching music), and primary education (the practice of music education at primary levels across disciplines). The participants were teaching different aspects of music including traditional folklore in China, Western music, and popular instruments (e.g., guzheng, dizi, erhu, and pipa). Musicology majors accounted for the largest proportion of participants in the questionnaire, accounting for 60%. Regarding their education, the participants had certificate (20.6%), high school diploma (27.6%), bachelor’s (26.2%), master’s (11.5%), and doctorate (14.1%). The teaching experience level of the teachers varied, too. Particularly, 246 had 1 year of teaching, 21 had 2 years of teaching, 13 had 3 years of teaching, 15 had 4 years of teaching, 11 had 5 years of teaching, and 34 had more than 5 years of teaching. The respondents attended the study based on convenience sampling and their freedom and confidentiality were ensured at the outset of the research.

### 3.2. Instruments

#### 3.2.1. Teacher commitment questionnaire

Allen and Meyer’s (1990) proposed questionnaire was used to measure this construct. It encompassed 18 items divided into three sub-components of affective commitment (6 items), continuance commitment (6 items), and normative commitment (6 items). The scale followed a 5-point Likert scale ranging from “1 = strongly disagree” to “5 = strongly agree.” As an example, “I would feel guilty if I left this university now” represents the first component of the employed scale. Concerning reliability, the results of Cronbach’s alpha indicated indices of 0.73, 0.81, and 0.79, respectively. In addition, McDonald’s omega reported indices of 0.71, 0.83, and 0.78, respectively.

#### 3.2.2. Teacher academic optimism questionnaire

To evaluate this variable, this study used Tschannen-Moran and Woolfolk Hoy’s (2001) questionnaire. It comprised 42 items, the answers to which can vary from 1 “Strongly disagree” to 5 “Strongly agree.” The questionnaire consists of three components, namely teachers’ sense of efficacy, teachers’ trust in parents and students, and individual academic emphasis. The reliability of the questionnaire components was calculated. The results indicated that the reliability index of each component for this inquiry was 0.79, 0.78, and 0.83, respectively. “I trust the parents’ of my students” is a sample question from the scale. In addition, McDonald’s omega reported indices of 0.78, 0.76, and 0.80, respectively.

#### 3.2.3. Teacher self-efficacy questionnaire

Concerning this variable, the researcher used Tschannen-Moran and Woolfolk Hoy’s (2001) self-efficacy questionnaire. It involved 24 items, each assessed on a 5-point Likert-type scale (1 = nothing, 5 = a great deal). The questionnaire comprises three different dimensions, including student engagement, classroom management, and instructional strategies. The overall reliability of this scale was 0.99 and the reliability of its sub-components was 0.77, 0.78, and 0.84, respectively. “How much can you do to get through to the most difficult students?” is an example from the scale. In addition, McDonald’s omega reported indices of 0.79, 0.75, and 0.82, respectively.

### 3.3. Data collection procedure

The study was conducted in the form of a booklet questionnaire including three separate scales related to each variable. After explaining the goal of the study and how the questionnaires ought to be responded, the researcher distributed an electronic version of the questionnaires among 340 Chinese music teachers. They had different demographic and educational backgrounds. The data collection was conducted in both English and Chinese lasting 2 months and completed in early March 2023. The data were collected from four universities in Henan Province, located in Xinxiang, Zhengzhou, and Xinyang. They were mostly teaching music in these universities, yet sporadically claimed to work in elementary and secondary schools, too.

It is essential to guarantee that the research was carried out with minimum risk to all parties engaged. Referring to autonomy, each participant was aware of the right to a free choice regarding participation or refusal to participate in the research, as well as withdrawal from the research at any time. The researcher also asked the participants to formally give their consent to participate. Additionally, the identity of all the participants was protected, and their right to privacy was guaranteed at all times. This also embraced their contact details, which were revealed to a third party without their consent. Finally, the collected data were protected and used exclusively for what they were gathered. Similarly, the principle of confidentiality was maintained at all stages of data collection and analysis. Additionally, the researcher assured the participants that their responses and data would be destroyed in an ethical manner after the completion of the research project. When the data were collected in the form of an Excel file, the researcher re-examined the responses for their accuracy and relevance. Then they were finalized for the subsequent statistical analyses using SPSS and Amos software.

### 3.4. Data analysis

The researcher used different statistical techniques to analyze the collected data. In doing that, the latest versions of SPSS and Amos software were utilized. To validate the scales, the original versions of the scales subjected to a confirmatory factor analysis (CFA). The scales were revised based on the results of CFA. The final versions of the scales were subjected to Cronbach’s alpha and McDonald’s omega to estimate the reliability of the instruments. Next, to answer the research question, the researcher used Linear Regression and SEM analysis to depict and test associational models of the three variables. Finally, appropriate statistical tables and figures were used to illustrate the final results.

## 4. Results

**Figure 3:**
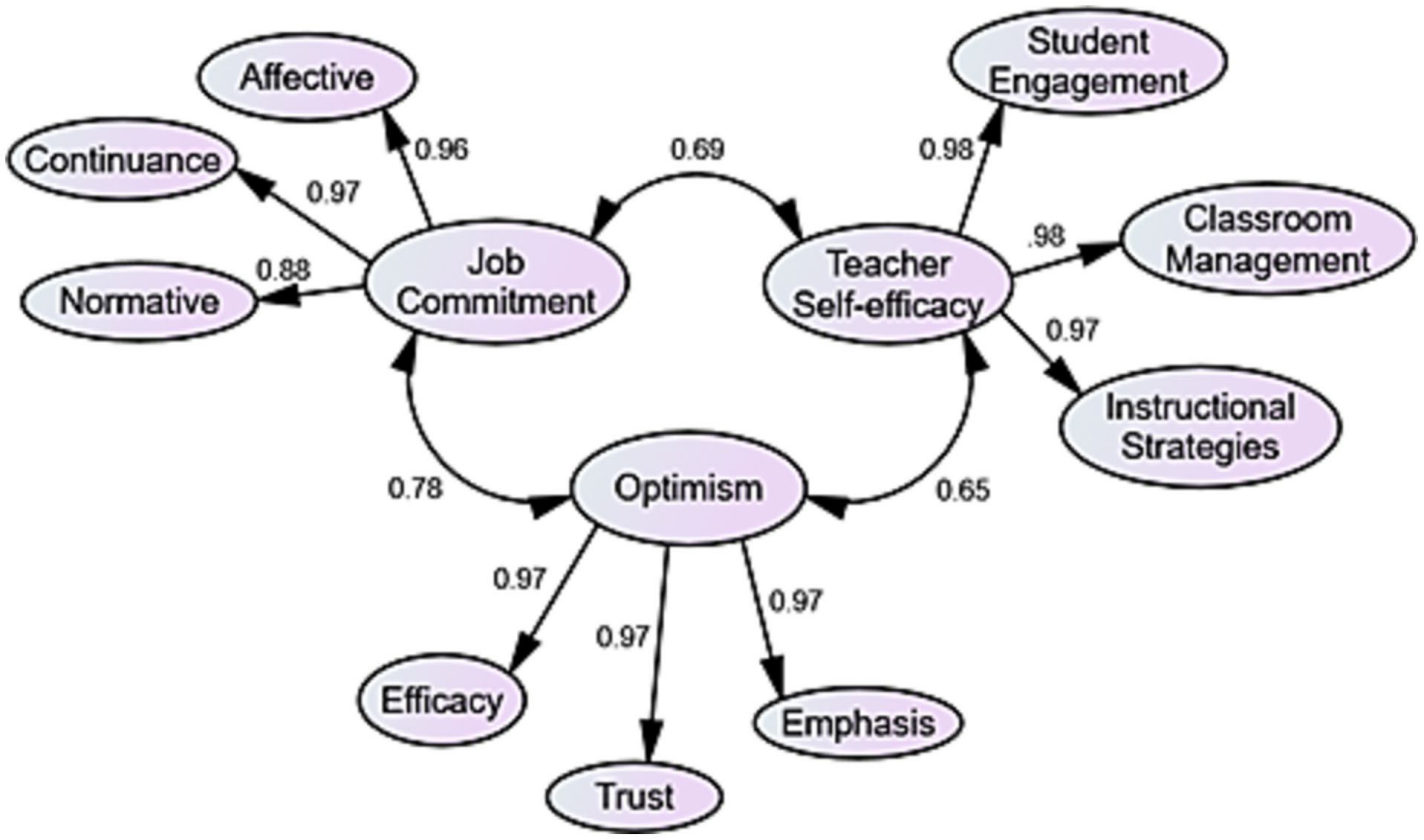
The final modified CFA model with standardized estimates.

To validate the employed scales in this study, CFA was performed through IBM SPSS Amos (Version 26). Based on previous studies and the review of existing literature, a three-factor model was proposed separately for each variable. To acknowledge the convergent validity of their relationship, CFA was run again. The initial model showed a good fit to the data (see Figure 3). Goodness-of-fit indices can be seen in Table 1.

**Table 1:** Evaluation of the CFA goodness of fit.

		Threshold			
Criteria		Terrible	Acceptable	Excellent	Evaluation
CMIN	9957.087				
DF	3,308				
CMIN/DF	3.010	>5	>3	>1	Acceptable
RMSEA	0.074	>0.08	<0.08	<0.06	Acceptable
GFI	0.935	<0.9	>0.9	>0.95	Acceptable
CFI	0.935	<0.9	>0.9	>0.95	Acceptable
PNFI	0.770	<0.5	>0.5		Acceptable
TLI	0.930	>0.9	>0.9	>0.95	Acceptable

In Table 1, the result indicated that five determiners are ratio of CMIN-DF, goodness-of-fit index (GFI), comparative fit index (CFI), Parsimonious Normed Fit Index (PNFI), Tucker–Lewis Index (TLI), and root mean square error of approximation (RMSEA). The model fit indices are all within specifications. Therefore, CMIN/DF is 3.010 (spec. ≤3.0), GFI = 0.935 (spec. >0.9), CFI = 0.935 (spec. >0.9), PNFI = 0.770 (spec. >0.5), TLI = 0.930 (spec. >0.9), and RMSEA = 0.074 (spec. <0.080).

The results of Table 2 show that composite reliabilities of the factors are acceptable (CR > 0.70). In other words, the model has achieved composite reliability. The values also demonstrate that the convergent validity of the factors reach to an acceptable value (AVE > 0.50) or the model has achieved convergent validity. Another requirement of convergent validity is factor loading more than 0.50. The results of factor loading are presented in Table 3. In addition, the results indicate that the model has achieved discriminant validity (the square root of AVE > inter-construct correlations).

**Table 2:** Composite reliability and discriminant validity of the factors.

	CR	AVE	MSV	MaxR(H)	Optimism	Teacher self-efficacy	Job commitment
Optimism	0.990	0.972	0.121	0.994	**0.986**		
Teacher self-efficacy	0.994	0.981	0.477	1.006	0.348	**0.990**	
Job commitment	0.956	0.878	0.477	0.969	0.278	0.691	**0.937**

Bold values are significance of the variable.

**Table 3:** Factor loading of the initial CFA model.

			Estimate	S.E.	C.R.	*p*
Teacher self-efficacy	<-->	Job commitment	0.694	0.072	9.423	0.000
Optimism	<-->	Teacher self-efficacy	0.651	0.065	5.740	0.000
Optimism	<-->	Job commitment	0.782	0.060	4.597	0.000
Affective	<---	Job commitment	1.008	0.054	18.628	0.000
Continuance	<---	Job commitment	1.072	0.051	21.033	0.000
Normative	<---	Job commitment	1.000			
Student Engagement	<---	Teacher self-efficacy	0.956	0.027	35.178	0.000
Classroom Management	<---	Teacher self-efficacy	1.001	0.021	47.040	0.000
Instructional Strategies	<---	Teacher self-efficacy	1.000			
Emphasis	<---	Optimism	1.000			
Trust	<---	Optimism	1.385	0.071	19.558	0.000
Efficacy	<---	Optimism	1.377	0.072	19.001	0.000
AF1	<---	Affective	1.000			
AF2	<---	Affective	1.011	0.044	23.093	0.000
AF3	<---	Affective	1.062	0.044	24.005	0.000
AF4	<---	Affective	1.062	0.039	27.374	0.000
AF5	<---	Affective	1.057	0.040	26.690	0.000
AF6	<---	Affective	0.822	0.065	12.606	0.000
CO6	<---	Continuance	1.000			
CO5	<---	Continuance	0.999	0.025	40.563	0.000
CO4	<---	Continuance	1.016	0.028	36.568	0.000
CO3	<---	Continuance	1.063	0.026	41.656	0.000
CO2	<---	Continuance	0.863	0.045	19.111	0.000
CO1	<---	Continuance	0.963	0.029	32.956	0.000
NO6	<---	Normative	1.000			
NO5	<---	Normative	0.883	0.040	22.194	0.000
NO4	<---	Normative	0.983	0.035	27.762	0.000
NO3	<---	Normative	0.967	0.036	26.554	0.000
NO2	<---	Normative	0.977	0.041	24.041	0.000
NO1	<---	Normative	0.920	0.036	25.429	0.000
SE1	<---	Student engagement	1.000			
SE2	<---	Student engagement	1.002	0.028	36.397	0.000
SE3	<---	Student engagement	1.014	0.027	38.240	0.000
SE4	<---	Student engagement	0.999	0.026	39.132	0.000
SE5	<---	Student engagement	0.994	0.026	38.812	0.000
SE6	<---	Student engagement	1.003	0.025	40.314	0.000
CM1	<---	Classroom management	1.000			
CM2	<---	Classroom management	0.993	0.022	46.084	0.000
CM3	<---	Classroom management	0.992	0.021	47.352	0.000
CM4	<---	Classroom management	1.017	0.020	50.756	0.000
CM5	<---	Classroom management	0.995	0.020	50.200	0.000
CM6	<---	Classroom management	0.981	0.021	47.844	0.000
SE7	<---	Student engagement	1.002	0.024	42.537	0.000
SE8	<---	Student engagement	1.017	0.024	43.001	0.000
CM7	<---	Classroom management	0.993	0.021	46.576	0.000
CM8	<---	Classroom management	0.978	0.022	44.427	0.000
IS1	<---	Instructional strategies	1.000			
IS2	<---	Instructional strategies	1.018	0.023	43.805	0.000
IS3	<---	Instructional strategies	0.990	0.023	43.170	0.000
IS4	<---	Instructional strategies	0.941	0.033	28.601	0.000
IS5	<---	Instructional strategies	0.996	0.022	45.427	0.000
IS6	<---	Instructional strategies	0.988	0.024	41.799	0.000
IS7	<---	Instructional strategies	1.005	0.022	46.311	0.000
EF6	<---	Efficacy	1.000			
EF5	<---	Efficacy	1.007	0.019	53.439	0.000
EF4	<---	Efficacy	1.036	0.019	53.802	0.000
EF3	<---	Efficacy	0.990	0.019	51.499	0.000
EF2	<---	Efficacy	1.011	0.019	53.641	0.000
EF1	<---	Efficacy	1.038	0.020	52.772	0.000
EF7	<---	Efficacy	0.990	0.018	54.645	0.000
EF8	<---	Efficacy	1.032	0.017	59.758	0.000
EF9	<---	Efficacy	0.962	0.021	45.608	0.000
EF10	<---	Efficacy	1.006	0.018	54.472	0.000
EF11	<---	Efficacy	0.905	0.024	38.235	0.000
EF12	<---	Efficacy	0.834	0.029	28.805	0.000
EF13	<---	Efficacy	0.973	0.022	44.155	0.000
EF14	<---	Efficacy	1.005	0.023	44.577	0.000
TR8	<---	Trust	1.000			
TR7	<---	Trust	0.936	0.021	45.366	0.000
TR6	<---	Trust	0.921	0.022	41.135	0.000
TR5	<---	Trust	1.010	0.019	54.353	0.000
TR4	<---	Trust	0.617	0.042	14.579	0.000
TR3	<---	Trust	0.994	0.024	42.145	0.000
TR2	<---	Trust	0.646	0.042	15.442	0.000
TR1	<---	Trust	0.742	0.035	21.051	0.000
TR9	<---	Trust	1.005	0.018	56.745	0.000
TR10	<---	Trust	0.952	0.021	44.841	0.000
TR11	<---	Trust	0.984	0.019	53.188	0.000
TR12	<---	Trust	0.806	0.030	27.288	0.000
TR13	<---	Trust	0.898	0.024	36.965	0.000
TR14	<---	Trust	0.568	0.045	12.609	0.000
EM8	<---	Emphasis	1.000			
EM7	<---	Emphasis	0.849	0.072	11.786	0.000
EM6	<---	Emphasis	1.057	0.067	15.862	0.000
EM5	<---	Emphasis	0.847	0.068	12.540	0.000
EM4	<---	Emphasis	0.977	0.068	14.474	0.000
EM3	<---	Emphasis	1.164	0.065	18.003	0.000
EM2	<---	Emphasis	1.017	0.066	15.438	0.000
EM1	<---	Emphasis	1.201	0.070	17.146	0.000
EM9	<---	Emphasis	1.366	0.068	19.950	0.000
EM10	<---	Emphasis	1.356	0.068	19.931	0.000
EM11	<---	Emphasis	1.146	0.067	17.158	0.000
EM12	<---	Emphasis	1.373	0.070	19.672	0.000
EM13	<---	Emphasis	1.348	0.069	19.572	0.000
EM14	<---	Emphasis	1.223	0.066	18.541	0.000

AF, affective commitment; CO, continuance commitment; NO, normative commitment; SE, student engagement; CM, classroom management; IS, instructional strategies; EF, sense of efficacy; TR, trust in parents and students; EM, individual academic emphasis.

The results of Table 3 show that almost all of the values are more than 0.50. It means that the model has achieved the convergent validity.

**Figure 4:**
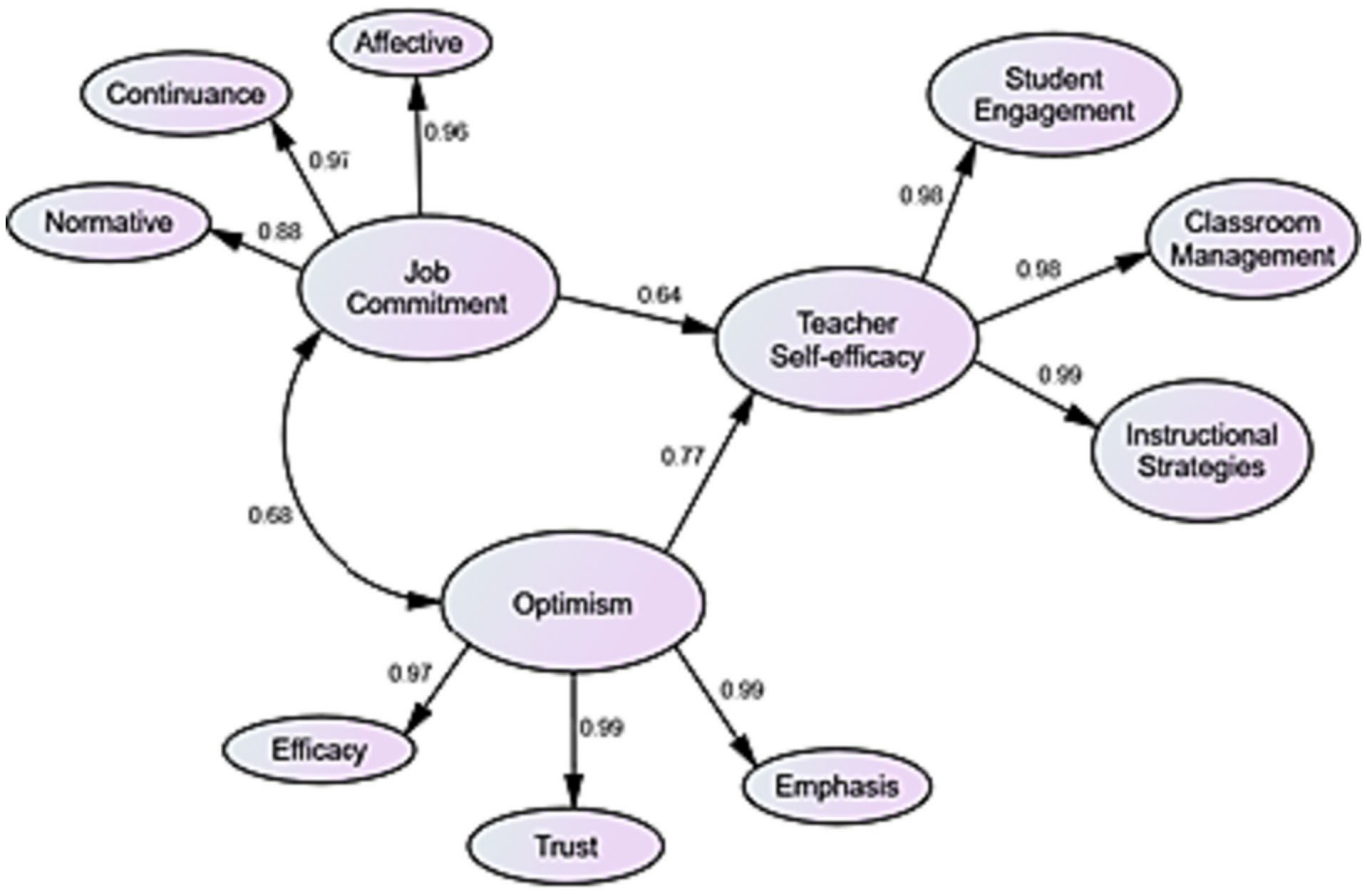
The final measurement model.

To answer the research question, Linear Regression was run in SEM. The estimation of such models has relied on covariance analysis methods, usually with the maximum likelihood (ML) estimator. The results of this analysis are presented in Table 4 and Figure 4.

**Table 4:** Results of linear regression analysis with SEM.

			Estimate	S.E.	C.R.	*p*
Teacher self-efficacy	<---	Job commitment	0.644	0.072	9.423	0.000
Optimism	--->	Teacher self-efficacy	0.771	0.065	5.740	0.000
Affective	<---	Job commitment	0.958	0.054	18.628	0.000
Continuance	<---	Job commitment	0.971	0.051	21.033	0.000
Normative	<---	Job commitment	0.879			
Student engagement	<---	Teacher self-efficacy	0.978	0.027	35.178	0.000
Classroom management	<---	Teacher self-efficacy	0.981	0.021	47.040	0.000
Instructional strategies	<---	Teacher self-efficacy	0.991			
Emphasis	<---	Optimism	0.988			
Trust	<---	Optimism	0.995	0.071	19.558	0.000
Efficacy	<---	Optimism	0.974	0.072	19.001	0.000

The results of Table 4 represent that music teachers’ self-efficacy can be strongly and favorably predicted by their job commitment (β = 0.64, *p* < 0.05) and optimism (β = 0.77, *p* < 0.05).

## 5. Discussion

This quantitative study examined the predictive role of Chinese music teachers’ commitment and optimism in their sense of self-efficacy. Using SEM and Regression analysis, the researchers found that job commitment could predict about 64% of changes in the teachers’ self-efficacy. This result concurs with Bandura’s (1997) SCT which regards self-efficacy as an outcome of numerous personal and environmental factors. Teachers’ job commitment is a sample personal factor that is affected by environmental factors and this interplay, in turn, determines music teachers’ self-efficacy beliefs. Additionally, this interaction is in line with the affective and normative dimensions of commitment, which highlight the emotional connection and obligation of a person to his/her profession. This form of attachment influences one’s behaviors, feelings, and practices, as well. The results are also empirically supported by de Vries (2013), who ran narrative research in music education and found that teachers’ self-efficacy beliefs are realized through their professional commitment. This correlation can be explained by the affective nature of both constructs in that a teachers’ emotional attachment to his/her job fosters strategic investment, enthusiasm, resilience, and dedication that play a critical role in self-efficacy beliefs. When a teacher is strongly committed to teaching, he/she constantly tries to increase his/her pedagogical abilities and certainty in implementing different methodologies. It seems that the participants had enough knowledge of PP and the interaction among various work-related and teacher-related factors. This might be due to Chinese teachers’ pre-service and in-service training, where the psychology of music education had been taught by experts. Another justification for this result can be the participants’ consideration of job commitment and attachment as a precondition for personal factors (i.e., self-efficacy). They had concerns about the job before their certainty in self-abilities. This can be attributed to their macro-view in music education.

Moreover, the results of this study indicated that optimism predicted about 77% of changes in the teachers’ self-efficacy. This statistical bond agrees with Hoy et al.’s (2006) conceptualization of academic optimism, which regards the sense of efficacy as a dimension of optimism. Likewise, this result is consistent with Seligman’s (2006) academic optimism theory, which underscored the contribution of optimism to the generation of several other outcomes in individuals (e.g., self-efficacy). Furthermore, the result augments SCT in that it adds a further approval to the idea that self-efficacy is affected by internal, individual variables, too. According to Bandura (1997), self-efficacy may emerge by a person’s physiological arousal in a job. Hence, it can be interpreted that optimism as an instance of such psycho-affective responses to teaching interacts with and stimulates music teachers’ self-efficacy. The result can be explained by Chinese teachers’ positive mentality and positive future outlook for music education that could foster their assurance in their abilities to teach music. This positivity might be developed in teachers during university education or other professional development programs. Another reason for this result could be the educational culture of China, which places emphasis on the psychology of teaching and learning. Correspondingly, teaching fine music, which is basically connected to one’s emotions and imagination, is best implemented in an emotion-based education. It also appears that the participants had a high emotional literacy regarding music education and its common psycho-emotional factors. That is why, they believed in the interplay of commitment, optimism, and self-efficacy. What is left unnoticed in this study is the possible intervention of demographics and educational profiles of the participants in the proposed model of association. This can be tested in future studies.

## 6. Conclusion and implications

The present study investigated the predicting role of music teachers’ job commitment and optimism in their self-efficacy. Based on the results, it can be concluded that like other disciplines, music education has a psycho-affective basis, which determines different areas of teachers’ work. To become sure of one’s skills to teach music, it is essential for teachers to be optimistic and committed to teaching, at first. When an instructor has a positive outlook of future and events in teaching music and is dedicated to it, he/she is more likely to experience self-efficacy compared to someone, who is dubious of the job and feels no attachment to it. In other words, music education is strongly connected to emotions and psycho-affective factors, which reciprocally interact to determine teachers’ behaviors and practices. This conceptualization is supported by PP, which sees academicians’ emotions tied to many other emotions. With these in mind, the current study is claimed to be advantageous to music teachers, trainers, and policy-makers. More particularly, the results can help music teachers understand the psycho-affective aspect of their profession and the interaction among three important factors involved in teaching music. They can use the results to develop a positive outlook of their profession and feel dedicated to what they are doing in a way that their inner states are positively triggered and enhanced. Moreover, music teacher trainers can utilize the results of this study and offer emotion-based training courses to music teachers, where practical techniques are taught to teachers to deal with emotions related to music education and pedagogy. They can elaborate on the principles and practices of PP in relation to music education in interactive courses with teachers. Similarly, policy-makers of music education can draw on the results can shift their decisions and plans from mere pedagogical concerns to psycho-affective considerations of teaching and learning fine music in China. They can envision and suggest practical plans to engage music teachers and learners through various motivators.

Despite these implications, this study has some limitations, especially its mere quantitative research design. Future research can be done using qualitative and mixed-methods designs in music education. Moreover, the data were collected from only a single context (i.e., China) and this requires care in interpreting and generalizing the results to other places. To compensate for this lack, further research is suggested to focus on cross-cultural studies on the interaction among commitment, optimism, and self-efficacy. Additionally, future researchers can compare and contrast different disciplines in light of the three constructs examined in this study. The differences between novice and experienced music teachers in light of the interplay of constructs is also recommended. Finally, the role of contextual and environmental factors in shaping teachers’ professional commitment, academic optimism, and self-efficacy has rarely been explored (Derakhshan et al., 2023). Future research is demanded to fill this gap.

## Data availability statement

The original contributions presented in the study are included in the article/supplementary material, further inquiries can be directed to the corresponding author.

## Ethics statement

The studies involving humans were approved by the Academic and Ethics Committee of Xinxiang University. The studies were conducted in accordance with the local legislation and institutional requirements. The participants provided their written informed consent to participate in this study.

## Author contributions

YD: Writing – original draft, Writing – review & editing.
